# Supplementary material for: Genome-wide association and expression quantitative trait loci in cattle reveals common genes regulating mammalian fertility
Source: Commun Biol. 2024 Jun 12;7:724. doi: 10.1038/s42003-024-06403-2 (PMC11169601; doi:10.1038/s42003-024-06403-2)
Supplement: Supplementary file 2 — Supplementary Information [file 42003_2024_6403_MOESM2_ESM.pdf]

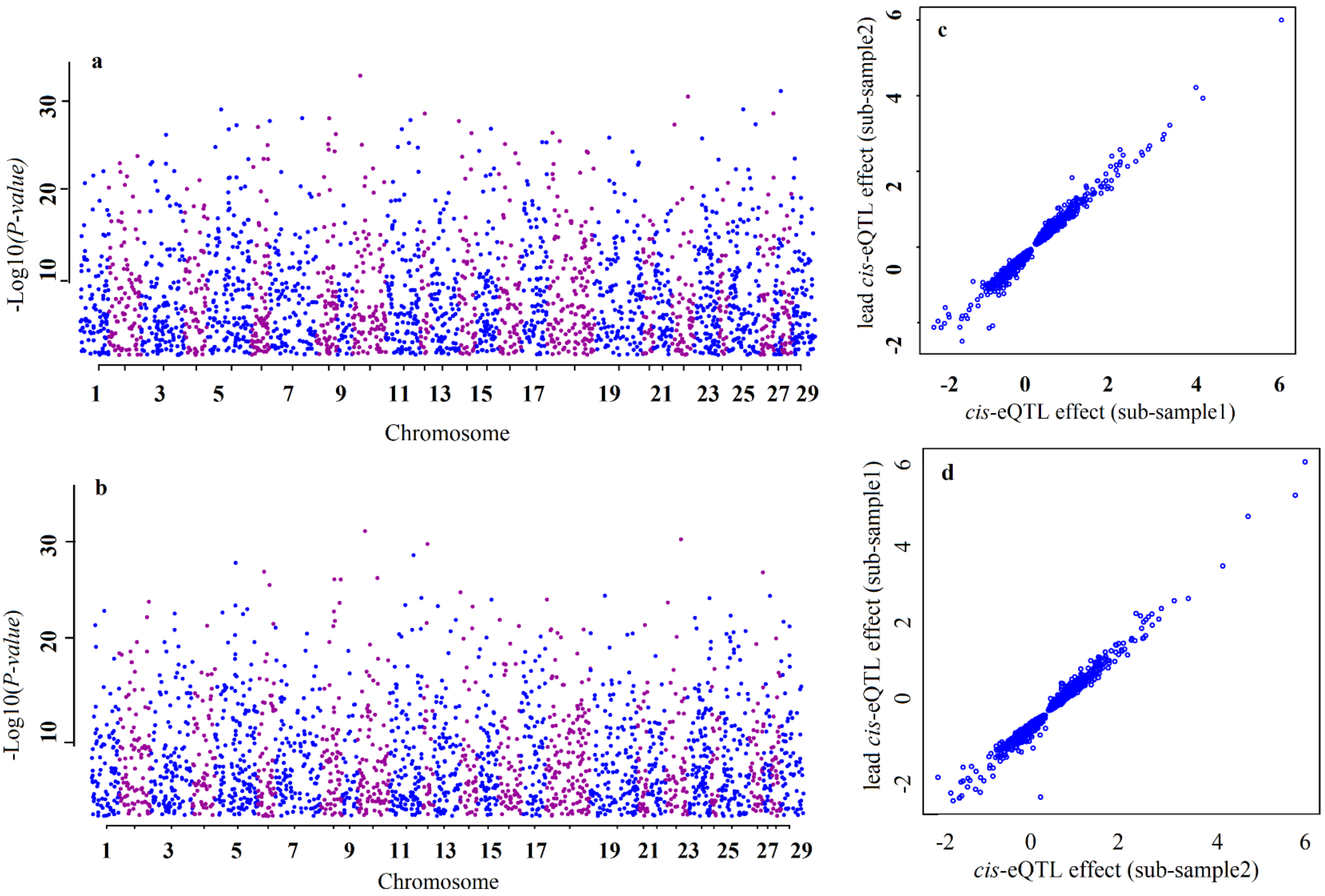

Supplementary Figure 1. Inter-validation of *cis*-eQTLs; Manhattan plot of lead *cis*-eQTL for eGenes (FDR<0.01) in half-sample2 (**a**) and half-sample1 (**b**), Consistency of effect estimates between pairs across half-sample2 (**c**), and half-sample1 (**d**)

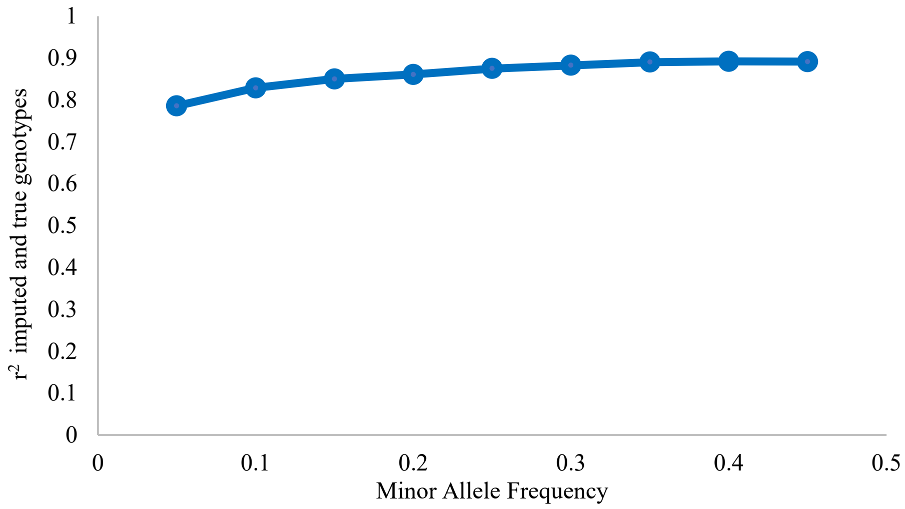

Supplementary Figure 2. Accuracy of imputation (correlation squared of true and imputed genotypes) to whole genome sequence data for the Brahman animals the 1000 bull genomes reference panel.

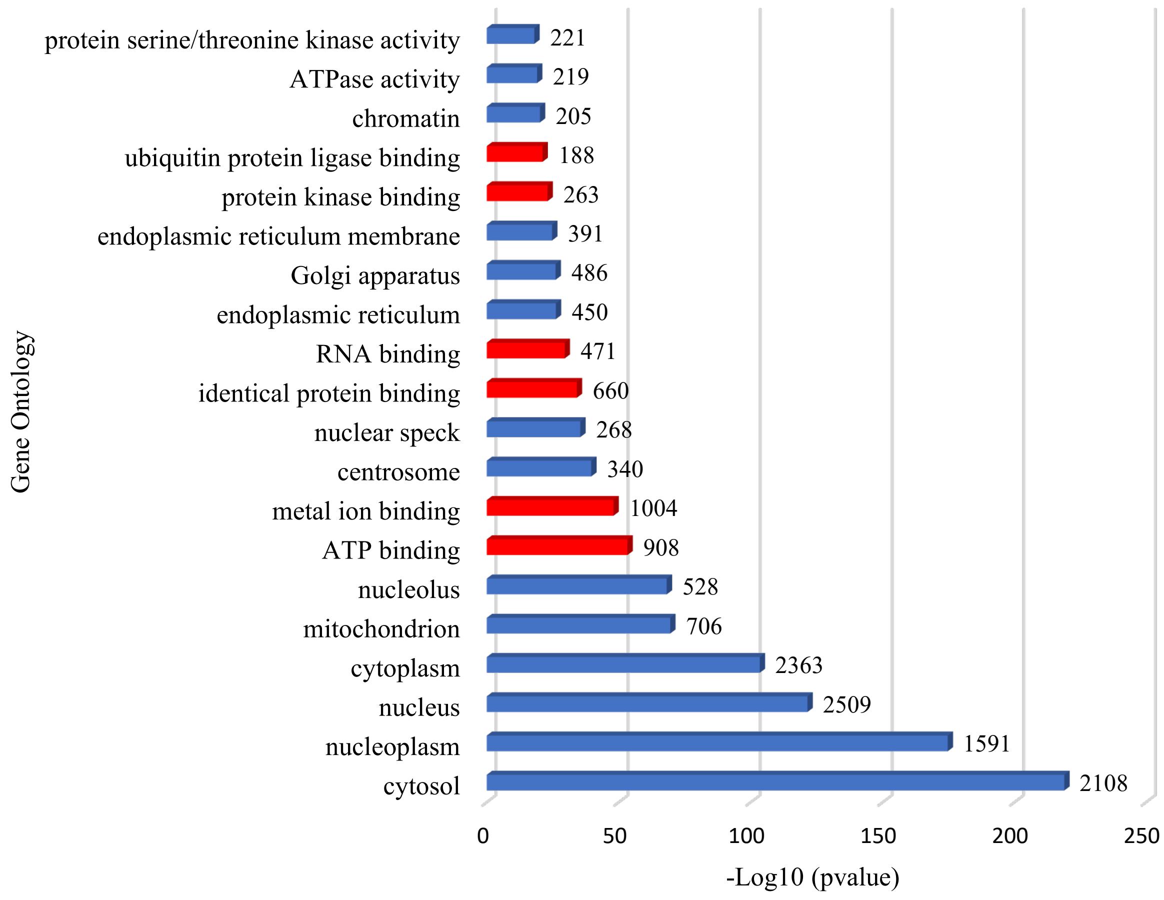

Supplementary Figure 3. The enrichment of top 20 most significant Gene Ontology terms, molecular function (red) and cellular component (blue), among the genes analysed. The number of genes associated with each Ontology term is indicated in front of the respective bar.
