# Supplementary material for: Genome-wide association and expression quantitative trait loci in cattle reveals common genes regulating mammalian fertility
Source: Commun Biol. 2024 Jun 12;7:724. doi: 10.1038/s42003-024-06403-2 (PMC11169601; doi:10.1038/s42003-024-06403-2)
Supplement: Supplementary file 3 — Description of Additional Supplementary Files [file 42003_2024_6403_MOESM3_ESM.pdf]

Description of Additional Supplementary Files

File name: Supplementary Data 1-14
Description: Additional data supporting the results.
